# Using technical assistance to bridge the gap between policy, research, and implementation

**DOI:** 10.3389/fpubh.2024.1347632

**Published:** 2024-06-12

**Authors:** Phillip L. Ealy, Crystal Tyler-Mackey, Kerri Ashurst, Misty Blue-Terry, Autumn Cano-Guin, Candi Dierenfield, Samantha Grant, Denae Harmon, Pamela B. Payne, Jennifer Wells-Marshall, Daniel F. Perkins

**Affiliations:** ^1^Clearinghouse for Military Family Readiness, The Pennsylvania State University, University Park, PA, United States; ^2^Department of Agricultural, Leadership, and Community Education/Virginia Cooperative Extension, Virginia Tech, Blacksburg, VA, United States; ^3^Family and Consumer Science Extension, University of Kentucky, Lexington, KY, United States; ^4^Youth, Families and Communities/Cooperative Extension, North Carolina Agricultural and Technical State University, Greensboro, NC, United States; ^5^4-H Youth Development, North Carolina State University, Raleigh, NC, United States; ^6^Extension 4-H, University of Florida, Gainesville, FL, United States; ^7^Center for Research and Outreach Lab, University of Minnesota Twin Cities, St. Paul, MN, United States; ^8^Human Development Family Science and Counseling and Extension, University of Nevada Reno, Reno, NV, United States; ^9^Alabama Cooperative Extension System, Alabama Agricultural and Mechanical University, Huntsville, AL, United States

**Keywords:** CYFAR, technical assistance, community programming, coaching, positive youth development

## Abstract

This case study on the Children, Youth, and Families At-Risk (CYFAR) Professional Development and Technical Assistance (PDTA) Center highlights a government-funded entity’s efforts to provide technical assistance to federal grantees of the CYFAR Sustainable Community Projects (SCP) grant program. The PDTA Center aligns with and supports components of an evidence-based system for innovation support. Through these components, the system provides targeted tools, training for CYFAR SCP grantees, dedicated technical assistance in the form of coaching, and quality improvement support through the evaluation of available program data.

## Introduction

In 2020, the United States Government Accountability Office (GAO) conducted a review of 10 federal grants, examining the technical assistance (TA) provided by three different agencies. The goal of the review was to assess how TA was being provided (e.g., format, approach) and how these agencies evaluated the TA support. The GAO found that TA varied across the different grants; however, they all contained an evaluation of the TA provided. The lessons learned from the evaluations were incorporated back into the agencies’ TA delivery to support grantees. Overall, the review found that TA can improve the management and performance of grantees served. Although not surprising, the lack of a clear definition of TA across the different federal grants was an important finding from the GAO’s review (1).

The GAO generally defines TA as “programs, activities, and services provided by federal agencies, nonprofit organizations, or another third party to strengthen the capacity of recipients and to improve their performance with respect to an inherent or assigned grant function” [(1), p. 3]. The GAO also noted that their findings were not generalizable “government-wide or to all grant programs at each of the three agencies” [(1), p. 2], thereby highlighting the need for further study and evaluation of TA within government grant programs.

In September 2023, the White House released a technical assistance guide outlining a list of over 100 programs designed to help state, local, Tribal, and territorial governments and other non-governmental partners implement programs in clean energy, infrastructure, and climate resilience (2). The support provided by these programs ranges from education and information support, direct TA provided by the program, and federal funding to acquire outside TA support. Education and information support include items such as websites, webinars, peer learning, and other information. Direct TA support is provided by federal staff or federally funded entities (e.g., subject matter experts). The third category of support allows for funds to be spent to hire internal staff or external entities to provide TA. Each of these programs provides one or a combination of all three types of support. The guide acknowledges that TA means something different depending on the user. Again, this supports the GAO (1) finding that there is no common definition within the U.S. government of TA or a standardization of what the TA support should entail. The U.S. Government uses various forms of TA to help organizations implement programs derived from policy as seen from the GAO review (1) and White House TA guide (2).

### Technical assistance

In their scoping review of peer-reviewed articles published between 2000 and 2020 in English on TA evaluation, Scott et al. (3) define TA as “an individualized, hands-on approach to capacity building in organizations and communities” [(3), p. 2]. The scoping review uncovered four main insights. The first insight was the need for a standardized definition of TA. The review recommended four defining features of TA, “[a]im is to increase capacity”; “[s]ervices target the systems-level (organization, community)”; “[s]upports are targeting and tailored”; and “[s]upports are provided by a subject matter expert or specialist” (p. 10). The second insight is the need for more rigorous evaluation given that a low rate of experimental designs or examinations of sustainability of TA outcomes was found. The third insight noted the need for more reliable and objective measurement of TA. Finally, Scott and colleagues pointed to the identification and usage of reporting standards for TA.

In 2012, Wandersman and colleagues published the evidence-based system for innovation support (EBSIS), where they outlined four support components. These components were tools, training, technical assistance, and quality assurance/quality improvement (p. 445). These four support components are the foundation of a continuous quality improvement process with the aim of capacity building. The EBSIS (4) has since been used to provide support to implement evidence-based programs (5–7). Proactive implementation support and TA can provide targeted support to community and project leaders by connecting them to expertise and resources that can address complex challenges (8–10).

This case study focuses on an implementation-support system titled the Children, Youth, and Families at Risk (CYFAR) Professional Development and Technical Assistance (PDTA) Center. The CYFAR PDTA Center is a four-year renewable grant-funded center through the United States Department of Agriculture (USDA) National Institute of Food and Agriculture (NIFA) with a mission to provide TA to support the USDA NIFA CYFAR Sustainable Community Projects (SCP) grant program (11). This Center is an implementation infrastructure that uses evidence-informed TA to support CYFAR SCP grantees (12) as they carry out community programming that focuses on children, youth, families, and communities that have historically been marginalized or underserved. Moreover, the components of CYFAR PDTA center are aligned with the EBSIS (4) components of tools, training, technical assistance, and quality assurance/quality improvement.

The purpose of this paper is to further the knowledge base on providing government TA support to grantees by examining the CYFAR PDTA Center implementation infrastructure showing how it fits the EBSIS (4) model and how it aligns with Scott et al.’s (3) defining features of TA. As mentioned before, the U.S. Government uses TA to implement government programs that were derived from policy. The TA provided has an opportunity to ensure programs are implementing policy driven programs through research.

## Context

### CYFAR SCP grant program

The CYFAR SCP grant program is designed to provide sustainable community programming in local communities and, especially, targets those who are most vulnerable. Grantees are awarded 5-year grants to accomplish the strategic objectives of supporting community educational programs and integrating the grant-funded programming into their land-grant institution’s Cooperative Extension System. Funded programming varies from each grantee with varying target audiences (e.g., immigrants, high school youth, middle school youth, families with grandparents as custodial guardians) and varying program types (e.g., healthy living, agriculture, workforce education, college preparation). The grant program has three major program components: being community-based, incorporating technology, and emphasizing sustainability (13).

In addition, USDA NIFA also awards a 4-year grant that focuses on proactive implementation support to CYFAR SCP grantees by the CYFAR PDTA Center (14) with the aim of addressing continuous quality improvement and capacity building of grantees. The CYFAR PDTA Center is a collaborative project comprised of the University of Minnesota, the Pennsylvania State University, coaches who represent multiple land-grant universities, and land-grant coordinators who represent Historically Black Colleges and Universities, Native American Tribal Universities and Colleges, and Hispanic Serving Institutions (15). The CYFAR PDTA Center was revised in 2013 when it transitioned from a liaison model to a coaching model as part of NIFA’s commitment to providing a more robust support system to CYFAR SCP grantees (12). The previous liaison model used liaisons “as intermediaries between USDA-NIFA administrators and grant recipients” [(12), p. 29]. The liaisons facilitated communications between USDA NIFA and the grantees. The coaching model provides each grantee with a dedicated coach. This allows for targeted TA support from a trained specialist. In the liaison model, the liaisons were a conduit for the grantor, USDA NIFA, whereas in the coaching model, the coach is there to support the grantee (12).

### How the CYFAR SCP grant program relates to public health

The USDA NIFA provides grant funding for various programs including the CYFAR SCP grant program (11). This grant program is a competitive, federal grant program involving land-grant universities and their Cooperative Extension Systems. The mission of CYFAR SCP grant program is to “marshal the resources of the Land-Grant and Cooperative Extension Systems to develop and deliver educational programs that equip limited resource families and youth who are at-risk for not meeting basic human needs with the skills they need to lead positive, productive, contributing lives” [(16), p. 6]. To accomplish this mission, CYFAR SCPs develop and implement educational programs for communities that are traditionally underserved and marginalized. These programs predominantly use trauma-informed practices to help underserved communities address social determinants of health, and these considerations include: education, employment, health systems and services, housing, income and wealth, the physical environment, public safety, the social environment, and transportation (17, 18). The CYFAR SCPs directly address Social Determinants of Health factors including nutrition, foodinsecurity, substance misuse, and obesity. Moreover, CYFAR SCPs target educational outcomes for youth are intended to increase resilience and self-efficacies among youth and caregivers with an emphasis on establishing health equity, defined as “personal agency and fair access to resources and opportunities needed to achieve the best possible physical, emotional, and social well-being” [(19), p. 742].

This focus on social determinants of health and trauma-informed practices to promote self-efficacies and resilience in traditionally marginalized groups is one of the hallmarks of the CYFAR PDTA center’s emphases on equitable implementation. Equitable implementation requires the integration of equity components, such as “explicit attention to the culture, history, values, assets, and needs of the community” [(20), p. 1] as a part of the quality program implementation (21).

## The CYFAR PDTA center implementation model

The CYFAR PDTA Center provides robust implementation support through evidence-informed implementation strategies and proactive TA. New CYFAR SCP grantees are added every year. The number added does not always match the number of grantees that are ending in the same year. However, there is a range of CYFAR SCP grantees each year, between 42–48. Each grantee is dynamic in their focus, approach, and needs they are addressing. For instance, some topics being addressed include youth workforce education programs, STEM programs, healthy living programs, college preparation programs, and parenting. Some grantees are joint, meaning they have partnered with another institution to provide similar programming that enables them to pool their different expertise. Grantees are in various phases of implementation. For example, grantees in their first year are focused on program planning and training while grantees in later years are focused on implementation and sustainability. Grantees are spread across the U.S. to include territories and vary in the target audience. Some focus on middle schoolers, some on high schoolers, some on multi-generational families, some on immigrants, some on rural populations, and some on urban populations. The support provided by the CYFAR PDTA Center aligns with the components of the EBSIS (4). The support has a standard baseline (e.g., tools, webinars, assigned coaches) that each CYFAR SCP grantee receives while maintaining flexibility to meet the individual needs of the grantee (e.g., type of training).

### Tools

Wandersman et al. (4) define tools as “informational resources designed to organize, summarize, and/or communicate knowledge” (p. 448). The CYFAR PDTA center develops and provides tools for the coaches to employ in support of the grantees to assist with implementation. The tools are designed to address incremental adjustments to programming. The website, cyfar.org, is where the tools are hosted. Two such tools include the logic model builder and survey builder. The logic model builder allows potential and current grantees the ability to format their project logic model to the NIFA standard required for application submission. The survey builder allows current grantees to build, deliver, print, and submit CYFAR common measure data. This data is then aggregated across the various sites by CYFAR PDTA center staff to provide an initiative-wide perspective on impacts. Other tools (e.g., fact sheets) are developed as required. When a need is identified the CYFAR PDTA Center team conducts research to develop tools that can be used by grantees for addressing risk and protective factors linked to Social Determinant of Health. Examples of tools include fact sheets on adverse childhood trauma, fatherhood, and families experiencing poverty; “how to” tools such as how to develop a program video and how to create an infographic; and checklists like the year one workbook that outlines key areas projects need to address during their first year of the five-year grant.

### Training

Wandersman et al. (4) define training as “a planned, instructional activity intended to facilitate the acquisition of knowledge, skills, and attitudes in order to enhance learner performance” (p. 449). Both general and program specific training opportunities are offered by CYFAR PDTA team. One of these opportunities is the use of webinars. Webinars are offered to CYFAR SCP grantees, their partners, and colleagues, and are specifically tailored to the needs each grant team has shared with their coaches. For example, webinars include topics such as implementation evaluation, forming partnerships with native communities, traumatic childhood experiences, and inclusion for immigrant youth in 4-H. A second support opportunity is the use of networking calls. These are offered to grant teams twice yearly and bring people together around commonalities, such as grant cohort year, audience focus, common curriculum used, or project positions (i.e., evaluators, principal investigators, site coordinators).

Another training opportunity to enhance the work of grantees is the annual CYFAR Professional Development Event (PDE). The PDE is an in-person gathering of all the grantee teams, the CYFAR PDTA Center team, and representatives from USDA NIFA. The PDE is focused on practical learning both general capacity building as well as program specific trainings through keynote speakers, breakout learning sessions, and networking time incorporated into the 2-day event for all grantees. The PDE also provides focused time for all grantees to meet with their coaches. An extra day is added for new grantees called New Grantee Orientation (NGO). NGO allows grantees an opportunity to learn about support and requirements regarding their award, become acquainted with the CYFAR PDTA team and their coach, and develop relationships with other new awardees who will be in the same 5-year cohort.

### Technical assistance

Wandersman et al. (4) define TA as “an individualized, hands-on approach to building an entity’s capacity for quality implementation of innovations, usually following training” (p. 449). A dedicated coach assigned to each CYFAR SCP grant following NGO. The coaching is a tailored component of the support provided to address the program specific training and support need by each grantee. The coaches offer proactive support as the main contact from the CYFAR PDTA Center to the CYFAR SCP grantee. CYFAR PDTA Center coaches are selected nationally from land-grant universities through an extensive selection process. The selection process focuses on identifying skillsets that coaches need to have to be successful based on the Coaching Masteries as established by the International Association of Coaching (IAC) (22). Selected coaches then go through an onboarding process that incorporates learning the following: IAC masteries (22), intricacies of the CYFAR PDTA Center, CYFAR PDTA Center tools (e.g., coaching notes, site visit manual), the evaluation process including the CYFAR Common Measures, program sustainability, and implementation tasks. Coaches are assigned to grantees, and a percentage of a coach’s time is allocated to that grantee. Coaches continue to receive professional development and training twice a month throughout their time with the CYFAR PDTA Center. Each coach has a one-on-one monthly call with the coaching coordinator, participates in the CYFAR PDE once a year, and participates in two in person team meetings.

The coach model is an integral part of the CYFAR PDTA Center. The overall goal of the coaching model is to promote successful programming and the demonstration of outcomes, especially sustainability. Based on the research, each new grant award is assigned a coach who works with them throughout their 5-year award. McCarthy et al. (10) found that coaching was more effective when there was consistency and collaboration, the technical needs of the grantee were met, and support often included face-to-face meetings. The CYFAR PDTA Center coaches engage in monthly Zoom calls with their assigned CYFAR SCP to discuss barriers, successes, planning, implementation, evaluation, and sustainability. This provides consistency, collaboration, and an opportunity to address the technical needs of the grant through face-to-face meetings between the coach and the grantee. This changes to Zoom calls every other month in the fifth year for the grantee.

Coaches partner with their CYFAR SCP grantees by listening to the unique needs, supporting the use of tailored resources, and actively incorporating employment of the TA tools into monthly coaching calls and site visits. Grantees are also provided with monthly newsletters and information sheets (e.g., implementation quality, recruitment strategies, and sustainability planning). The coach travels to the grantee and completes an in person site visit with the grant team, their administrators, and community partners before the end of the first year of the grant to help assemble the initial program-implementation pieces, bolster relationships, and troubleshoot barriers to success. The coach then travels for a second site visit with the team during the 3rd year of their grant to again meet with numerous groups who are involved with the grant, garner administrative support, and observe programming. During the 5^th^ year of the grant, the calls are heavily focused on the sustainability of the program and the completion of all the remaining grant requirements.

The coach also spends focused time with each grant team once per year while all grantees meet at the annual PDE. In between scheduled virtual and in person contacts, the coach serves as a support resource for the grant teams in various forms: advocate, champion, and objective listener. Coaches enter notes into the CYFAR PDTA Center’s online reporting system following monthly calls and site visits with assigned grantees. These notes help the funder generate a collective knowledge base of site implementation practices and serve as a mechanism for identifying resources that are requested or that need to be created for CYFAR SCP grantees. A study by Olson et al. (12) shows that using coaches within the CYFAR TA-support model has a positive impact on CYFAR SCP grantees.

### Quality assurance/quality improvement

Wandersman et al. (4) define quality assurance/quality improvement as “an integrative process for identifying current levels of quality and for improving quality performance” (p. 453). The CYFAR PDTA Center uses a rich data-Research Topic process to better understand and support the needs of CYFAR SCP grantees to improve the quality of support provided. Data are also used to share information about the impact of CYFAR SCP programs to NIFA leadership and other financial stakeholders. Cross-site evaluation provides a way to build grantee confidence and illustrates that, although there are diverse content areas for CYFAR SCPs, there are core features for every program—programs are high quality and focus on building strong individual capacity among participants. The results also provide grantees with information on process outcomes like engagement and program quality. Summative outcomes assess resilience and life skills, which are two outcomes program participants should develop regardless of the individual CYFAR SCP program.

Primary data include the following:

- Common measures: The CYFAR Common Measures are a set of reliable, valid measures on dosage, engagement, program quality, life skills, and resilience (23).
- Sustainability data: Sustainability surveys use the Program Sustainability Assessment Tool (PSAT) from Washington University.^1^ The PSAT uses eight measurement domains: environmental support, funding stability, partnerships, organizational capacity, program evaluation, program adaptation, communications, and strategic planning.
- Coach-Principal Investigator (PI) survey data: The Coach/PI survey is aligned to the IAC masteries (22) and is used to gauge PI perceptions of coaching practice. Survey results are used to plan targeted professional development for coaches.

#### Common measures

CYFAR SCPs collect common data from all child, youth, and adult participants and provide deidentified data to the CYFAR PDTA Center. The CYFAR Common Measures are valid, reliable evaluation measures that have research support (23). Beginning in 2020, CYFAR SCPs began collecting a streamlined set of common measures, including resilience and life skills as outcome measures.

The PDTA Center analyzes the role of youth program quality and engagement components in youth outcomes to guide program staff in their quality improvement efforts to support youth positive development. In a multiple regression model, program quality component scores significantly predicted youth outcome change scores. Positive social norms predicted change in critical thinking, decision making, and personal values, and opportunities for skill building predicted change in personal values and social conscience (24). For example, the positive social norms component had a significant effect (*β* = 0.33, *p* < 0.05), and skill-building opportunities showed a significant effect (*β* = 0.29, p < 0.05) on critical thinking and decision-making skills.

Additionally, in a multiple regression model, youth program engagement (interest and investment) predicts resilience and critical thinking across 10 CYFAR youth programs nationwide. None of the other measures of engagement (duration, intensity, and breadth) were associated with changes in resilience scores. One explanation is that programs of varying durations and intensities can foster youth investment in the program, leading to resilience. Duration was a predictor of change in critical thinking scores (*β* = 0.18, *p* < 0.05), suggesting that there may be specific skills along the thriving trajectory for which dosage is more important (25).

Using hierarchical multiple regression analyses, the PDTA Center assessed the role of program quality components of skill-building, leadership opportunities, positive adult relationships, as well as the additional component of equitable climate, in increased civic engagement skills. Results indicate that youth participating in positive youth development programs report a significant increase in civic engagement skills [*t*(286) = 4.42, *p* < 0.001, d = 0.23]. Results further indicate that skill-building opportunities (*β* = 2.41, *p* = 0.001) and equitable climate (*β* = 1.54, *p* = 0.034) are associated with higher civic engagement scores among youth (25).

With additional data from CYFAR SCPs, the CYFAR PDTA Center intends to explore structural equation models using the full complement of common-measures data. In addition, CYFAR PDTA Center staff will further examine program-process data to evaluate CYFAR SCPs’ program quality and further establish that measure in the literature.

#### Sustainability

The CYFAR PDTA Center collected data using the PSAT as part of the CYFAR PDTA Center’s efforts to evaluate and improve sustainability of partner CYFAR SCPs. The PSAT is a 40-item survey that is answered by project staff and separated into eight domains: environmental support, funding stability, partnerships, organizational capacity, program evaluation, program adaptation, communications, and strategic planning. The PSAT has high reliability in assessing the sustainability of social programs (26).

The sustainability data were analyzed and compared to former CYFAR SCP grantee sustainability data. In addition to average domain scores, one of three sustainability levels was assigned to former grantees based on the self-reported extent of program activities sustained since the end of CYFAR funding. The data showed that former grantees who reported expanded activity had higher sustainability scores than former grantees who reported reduced activity. The domains with the largest gaps in average scores were organizational capacity, communication, and strategic planning. These findings suggest that, within the PDTA Center’s pool of former grantees, these domains had the highest relative importance in determining post-CYFAR funded activity levels, which can indicate overall sustainability.

#### Coach-PI survey

A survey was distributed to CYFAR SCP grantees in fall 2023 to understand their perception of the coaching provided by PDTA. Grantees were asked about their experiences of being coached (e.g., trust, communication, disclosure), expectations around coaching, and coach characteristics (e.g., listens, supports) throughout the grant process. The questions were tailored around the IAC’s nine Coaching Masteries (22). Thirty-one of 38 grantees (82%) submitted a response to the survey for a total of 36 respondents (some grants have multiple PIs). Over 90% of participating grantees reported that they agreed or strongly agreed that coaching expectations were clear, their coach affirmed their team’s potential, they were comfortable sharing concerns and successes with their coach, and their coach helped them devise solutions to address challenges. The results of the Coach-PI survey are used to help direct coach training and development by identifying trends in either the coaching cadre or individual coaches.

Grantees were asked an open-ended question about their coach’s strengths. Thirty-five of the 36 respondents made comments regarding their coach. Responses included “very supportive of the project work,” “excellent listener and communicator, very friendly: helps build confidence in programming directions,” and “they are very approachable and knowledgeable, we feel very comfortable asking for help or direction to achieve successful outcomes.” Another respondent stated:

They are an amazing coach, and we are fortunate to have them on our side. This was my first CYFAR grant, so things felt quite overwhelming at first, but they were always encouraging and made it clear that they were in our corner to support us every step of the way. I couldn’t ask for anything more in a coach.

These comments highlight the value and impact coaches had on the CYFAR SCP grantees.

#### 2019 CYFAR SCP cohort

The 2019 cohort of CYFAR SCPs were provided an additional survey that asked about sustainability post-CYFAR funding and CYFAR PDTA Center support. Sixteen out of the 20 CYFAR SCP grantees in the 2019 cohort that responded. All 16 grantees believe they will sustain programming in some form post-CYFAR grant funding. The 2019 cohort was asked what the CYFAR PDTA Center is doing well. Leading responses included coaching, providing the PDE, being accessible, and offering networking calls. The level of TA support that is provided by the CYFAR PDTA Center through internal (e.g., Coach PI-Surveys, Sustainability Surveys) and external (e.g., scholarly articles) research vastly increases the likelihood that grantees will achieve program sustainability and improves implementation. The results of surveys (i.e., Coach-PI survey, PSAT, cohort surveys) indicate that the support provided by the CYFAR PDTA Center is perceived to be effective. However, the data lacks the rigor to determine the extent of effectiveness and generalizability. Nevertheless, the preponderance of data for more than 8 years indicated progress toward increases in outcomes being examined and in CYFAR SCP grantees overall satisfaction and success as measured by program sustainability beyond the CYFAR funding.

## Discussion

The CYFAR PDTA Center serves as the TA component that bridges the gap between USDA NIFA policy and CYFAR SCP grantees’ implementation of community-based programs using research to develop tools, provide training, provide TA support, and constantly conduct quality improvement. While the CYFAR PDTA Center did not intentionally follow the EBSIS (4) components, the PDTA support aligns with these components as outlined above by using research and practitioner feedback to improve the tools, training, technical assistance, and quality assurance/quality improvement of the TA support provided. The CYFAR PDTA Center consistently uses research to build tools and resources, implement coach training, and refine TA support for grantees. Having access to dedicated TA support is a key factor in the level of success of programming. The coaching support that a site receives is backed by a whole TA team who supports the sites and provides direct support to the coaches through training and development, research, evaluation, and specialized technical support (e.g., CYFAR common measures, Logic Model Builder). From the initial new grantee orientation, grantees understand that they will have comprehensive TA support for the duration of the 5-year grant cycle. Five years of dedicated funding allows grantees to deliberately build and implement programming within their communities. Moreover, with the support of the CYFAR PDTA Center team, grantees develop and implement a sustainability plan starting in year one. Having dedicated support that incorporates the components of the EBSIS (4) over five years aids in successful programming.

As mentioned before, a main insight Scott et al. (3) identified is a need for a standard definition for TA. The GAO (1) and the U.S. White House (2) also noted the lack of a standard definition for TA. Again, Scott et al. (3) did outline some defining features of TA. The CYFAR PDTA Center adheres to Scott et al.’s (3) defining features of TA. The CYFAR PDTA center aims to increase the capacity of CYFAR SCP grantees to implement community programming. The services are targeted to increase system-level support (e.g., program staff, institution extension, community relations). Support is tailored to each grantee (e.g., networking calls based on project type). Finally, support is provided by subject matter experts and specialists (e.g., assigned coaches and evaluation support specialists). Using Scott et al.’s (3) defining features of TA, a proposed standardized definition for TA would be tailored support provided by a or a group of subject matter expert(s) or specialist(s) that is designed to increase organizational capacity to improve organizational effectiveness and performance.

With regards to Scott et al.’s (3) second insight, the support involves evaluation, albeit not as rigorous as an experimental design. The CYFAR PDTA Center is moving toward this goal with plans for longitudinal evaluation of components of the TA provided (e.g., site visits). Efforts have also been made to standardize an exit interview protocol for year-five grantees. This, in conjunction with other evaluation efforts that have already begun (e.g., PSAT), will be able to address the impact of the TA support more definitively.

The CYFAR PDTA Center has implemented reliable measures (e.g., resiliency, PSAT) and continues to develop additional measures (e.g., Coach-PI survey) to measure and improve the TA support provided. The measures, along with the reporting standards (e.g., Annual Report, Site Visit Report) in place, the CYFAR PDTA Center adheres to Scott et al.’s third and fourth insights outlined in their scoping review.

The CYFAR PDTA Center’s continuous quality improvement serves as a model for implementing TA support to U.S. government programs that have been funded through policy decisions such as the CYFAR SCP grant program. Clearly defining TA and holding TA support to EBSIS (4) components allows for TA to support programs using research to implement with quality. In the research-practice-policy partnership, USDA NIFA serves as the policy arm, while the CYFAR SCP grantees understand the research of their particular program, and the CYFAR PDTA Center serves as the lead component on helping the CYFAR SCP grantees practice, or implement, programing with quality.

## Constraints

While there is evidence that TA is working, the lack of a formal study conducted that directly examines the impact TA provided by the CYFAR PDTA Center ultimately led to the development of this case study, which evaluates the effects of the CYFAR PDTA Center on the various CYFAR SCP grantees. The work that the CYFAR PDTA Center engages in is evidence-informed. Having additional resources to conduct a longitudinal study in the future could determine if the CYFAR PDTA Center model could serve as an evidence-based resource.

Another major challenge to evaluating the CYFAR PDTA Center’s TA support, along with any TA support, is that there are an exorbitant number of variables to account for. However, this is where The CYFAR PDTA Center could be unique. While the TA support is singular in focus with regard to supporting CYFAR SCP grantees, these grantees and their activities and needs are varied in number, type, and target audience. There is also built-in longevity due to the grant being 5 years. Overcoming the challenges of resources and evaluation design with so many variables to account for could lend to a valuable study that would add value to the field of TA.

## Data availability statement

The data analyzed in this study is subject to the following licenses/restrictions: For CYFAR use only. Requests to access these datasets should be directed to DH, harmo312@umn.edu.

## Author contributions

PE: Conceptualization, Project administration, Writing – original draft , Writing – review & editing. CT-M: Writing – original draft. KA: Writing – original draft. MB-T: Writing – original draft. AC-G: Writing – original draft. CD: Writing – original draft. SG: Data curation, Writing – original draft. DH: Data curation, Writing – original draft. PP: Writing – original draft. JW-M: Writing – original draft. DP: Writing – original draft, Writing – review & editing.

## Footnotes

^1^ https://sustaintool.org/psat/
